# Biomechanical Analyses of Porous Designs of 3D-Printed Titanium Implant for Mandibular Segmental Osteotomy Defects

**DOI:** 10.3390/ma15020576

**Published:** 2022-01-13

**Authors:** Yen-Wen Shen, Yuen-Shan Tsai, Jui-Ting Hsu, Ming-You Shie, Heng-Li Huang, Lih-Jyh Fuh

**Affiliations:** 1 School of Dentistry, China Medical University, Taichung 404, Taiwan; a2312830@ms28.hinet.net (Y.-W.S.); sunshine120807@gmail.com (Y.-S.T.); jthsu@mail.cmu.edu.tw (J.-T.H.); eric@mail.cmu.edu.tw (M.-Y.S.); 2 Department of Bioinformatics and Medical Engineering, Asia University, Taichung 413, Taiwan; 3 x-Dimension Center for Medical Research and Translation, China Medical University Hospital, Taichung 404, Taiwan

**Keywords:** mandibular segmental defect, 3D-printed porous titanium mandibular implant, pore shape, pore size, finite element analysis, strain gauge in vitro experiment, stress, strain

## Abstract

Clinically, a reconstruction plate can be used for the facial repair of patients with mandibular segmental defects, but it cannot restore their chewing function. The main purpose of this research is to design a new three-dimensionally (3D) printed porous titanium mandibular implant with both facial restoration and oral chewing function reconstruction. Its biomechanical properties were examined using both finite element analysis (FEA) and in vitro experiments. Cone beam computed tomography images of the mandible of a patient with oral cancer were selected as a reference to create 3D computational models of the bone and of the 3D-printed porous implant. The pores of the porous implant were circles or hexagons of 1 or 2 mm in size. A nonporous implant was fabricated as a control model. For the FEA, two chewing modes, namely right unilateral molar clench and right group function, were set as loading conditions. Regarding the boundary condition, the displacement of both condyles was fixed in all directions. For the in vitro experiments, an occlusal force (100 N) was applied to the abutment of the 3D-printed mandibular implants with and without porous designs as the loading condition. The porous mandibular implants withstood higher stress and strain than the nonporous mandibular implant, but all stress values were lower than the yield strength of Ti-6Al-4V (800 MPa). The strain value of the bone surrounding the mandibular implant was affected not only by the shape and size of the pores but also by the chewing mode. According to Frost’s mechanostat theory of bone, higher bone strain under the porous implants might help maintain or improve bone quality and bone strength. The findings of this study serve as a biomechanical reference for the design of 3D-printed titanium mandibular implants and require confirmation through clinical investigations.

## 1. Introduction

Mandibular segmental defects typically result from jaw trauma, tumor resection, or osteoradionecrosis. If not treated early, they can compromise patients’ ability to talk and eat. Common clinical treatments for segmental defects of the mandible include autologous bone grafting and bone plate reconstruction. Autologous bone grafting typically involves one of two types of implants, nonvascularized bone grafts and vascularized bone flaps, which commonly use the fibula, ilium, or ribs as donor sites [1,2]. Patients indicated for such procedures typically undergo a preoperative evaluation through which factors such as age and the location and severity of the defect are considered [3]. The defect severity can be clinically used to determine the method of transplantation. If the area of a segmental mandibular defect is small, a nonvascularized bone flap graft or a bone plate is employed. If the area is large, vascularized bone flap transplantation is preferred [4,5]. However, patients with large-scale bone defects may still cause possible peri-implant bone loss [6,7], even if they received the treatment of bone grafting mentioned above and dental implant.

With advances in medical image processing, computed tomography (CT) and magnetic resonance imaging have become more precise and can even reach the micro-level to analyze material structure and new bone formation processes [8]. With the aid of software, the internal structure of the human body can now be displayed as three-dimensional (3D) images, assisting physicians in making more accurate presurgical evaluations. These images can be directly read by 3D printers after they have been converted into the Surface Tessellation Language file format; thus, the popularity of 3D printing technology in medicine is growing [9,10,11].

The use of 3D printing technology to make a solid model of a patient’s bones as an aid in preoperative and postoperative assessment is increasing. Employing 3D printing technology in this context can reduce both the time and cost of the operation [10,12,13]. Notably, patient-specific 3D-printed implants have passed the in vitro and animal testing phases and have entered clinical settings [14,15]. For example, 3D printing has been used in recent years in craniofacial and maxillofacial surgery to fabricate patient-specific Ti cranial or oral implants [16,17,18].

The inspiration for this research was a study by Huang et al. (2018) [19]. Finite element analysis (FEA) of their self-designed metal mandibular implants for segmental mandibular defects revealed that, regardless of changes in the shape, thickness, or length of the implants, high stress occurred at specific locations. These included the position of the base post close to the abutment as well as the wing plate used to connect the implant to the mandible. The purpose of this study is to optimize the biomechanical performance of 3D-printed metal mandibular implants with prosthetic bases for segmental mandibular defects wherein the design parameters of circular and hexagonal pores were applied. FEA and in vitro experiments were conducted to investigate how the porous structure affected the stress–strain distributions of the implant body and of the surrounding bone.

## 2. Materials and Methods

### 2.1. 3D Computer Model Establishment

The study protocol was approved by the Institutional Review Board (IRB) of China Medical University Hospital (approval no. CMUH108-REC3-174). Specifically, permission was granted for the acquisition of cone beam CT images of the mandible of a patient with oral cancer (Planmeca ProMax 3D Max, Planmeca USA, Hoffman Estates, IL, USA). The voxel resolution of the images was 0.65 mm. The images were imported into the medical image processing software Mimics (Materialise, Leuven, Belgium) to identify the contours of cortical bone and spongy bone through the separation of grayscale threshold values. Subsequently, these contours were saved in the Initial Graphics Exchange Specification file format and imported into a reverse engineering software (Geomagic Inc., Morrisville, NC, USA) to establish a primary model of mandibular reconstruction. After the uneven surface of the model was smoothed, the model was imported into a computer-aided design software (Solidworks, Dassault Systemes SE, France), with the *x*-axis corresponding to the tongue and the buccal direction, the *y*-axis corresponding to the mesial and distal direction, and the *z*-axis corresponding to the bite direction.

**Figure 1:**
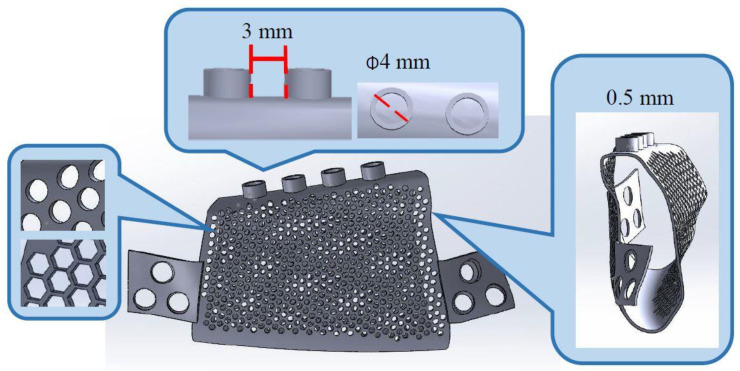
Design features of the metal mandibular implant.

After the right malignant tumor bone from the patient’s 3D mandibular model was removed programmatically, the main body and wing plate of the mandibular implant were designed according to the characteristics of the defect site (Figure 1). First, the implant conformed to the anatomical shape of the mandible, and the main part of the implant was hollow and 0.5 mm thick. The hollow body provides space for bone growth under bone grafting. Second, round and hexagonal pores with diameters of 1 and 2 mm were used. The porous design was intended to increase blood flow both inside and outside the implant, thereby promoting healing after bone grafting. Furthermore, it reduced the weight of the implant [20]. The abutment base was situated 3 mm above the implant. The abutment base can be connected to the crown to restore the patient’s occlusal function (Figure 1).

Two wing plates were set on both sides of the implant. Each wing plate had three screw holes to secure the plate to the mandible, and the implant can be positioned at the mandibular resection. To achieve a satisfactory fit between the jawbone surface and each wing plate, a model of the curved surface of the jawbone was imported into the Rhinoceros 4 software (Robert McNeel & Associates, Seattle, WA, USA).

**Figure 2:**
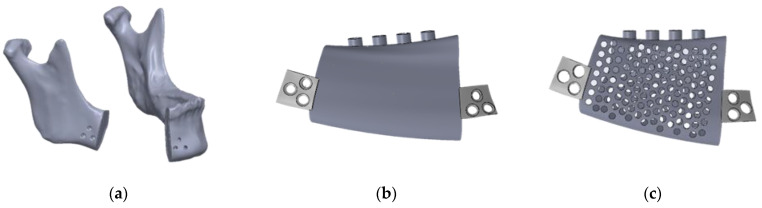

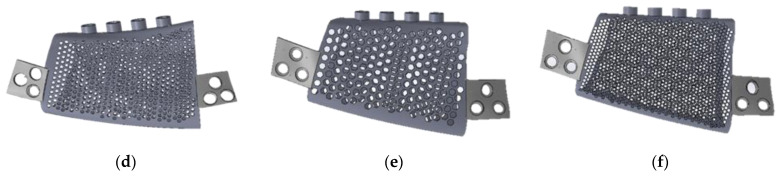
(**a**) Mandibular model and metal mandibular implants (**b**) without pores, (**c**) with circular holes 2 mm in diameter, (**d**) with circular holes 1 mm in diameter, (**e**) with hexagonal holes 2 mm in diameter, and (**f**) with hexagonal holes 1 mm in diameter.

Five mandibular implant models (Figure 2) were designed. The control model had no pores. As for the other four models, they featured round holes 2 mm in diameter, round holes 1 mm in diameter, hexagonal holes 2 mm in diameter, and hexagonal holes 1 mm in diameter. After the five models were combined with the model of the mandible, a finite element analysis was performed.

### 2.2. FEA

After the five 3D models were imported into the Ansys software (version 2020 R2; Ansys, Inc., Canonsburg, PA, USA), the second-order tetrahedral elements were used for meshing to create a 3D model for FEA.

The material parameters are presented in Table 1 [19]. Settings of linear elastic, isotropic, and homogeneous materials were applied to the cortical bone, cancellous bone, mandibular implants, and screws. The displacements of the condyles in the *x*, *y*, and *z* directions were fixed at zero. The bone–screw interface was bonded, as was the bone plate–screw interface.

**Figure 3:**
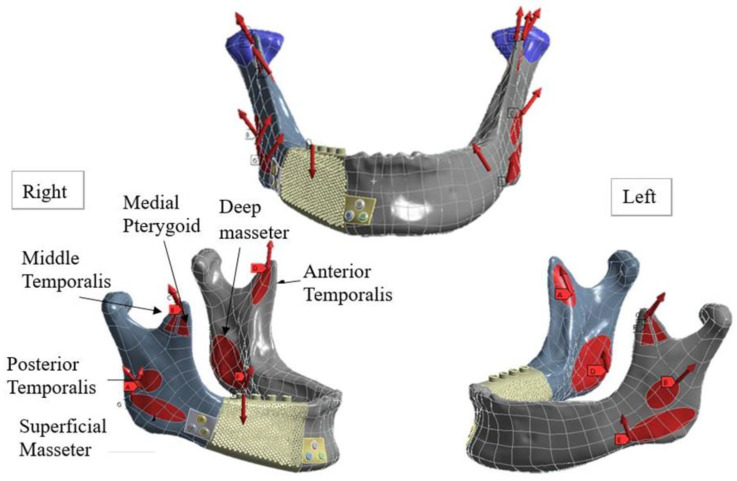
Loading conditions.

Two occlusal modes were employed for load simulation. Specifically, the three primary muscles used in mandibular movement, namely the masseter, medial pterygoid, and temporalis, were set to the right unilateral molar clench (RMOL) and right group function (RGF) modes (Figure 3) [21,22]. The loading conditions are presented in Table 2.

### 2.3. 3D Printing and In Vitro Experiments

To evaluate the effects of the porous design on the biomechanical performance of the titanium mandibular implant, experiments were conducted on the control implant and on the implant with 1 mm circular holes. The Objet 500 Connex3, a light-curing 3D printer (Stratasys, Eden Prairie, MN, USA), was employed. In order to increase the anatomical accuracy of the mandibular bone model in this experiment, two types of resin with differing densities, specifically VeroWhitePlus (Stratasys) and TangoPlus (Stratasys), were used to fabricate the compact and cancellous bone of the mandibular bone model, respectively.

**Figure 4:**
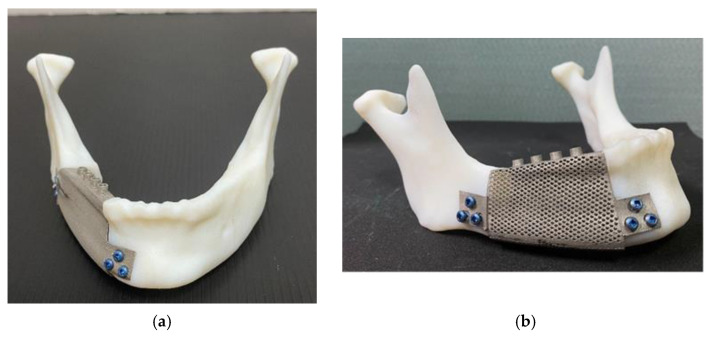
(**a**) Frontal view of the control sample; (**b**) lateral view of the experimental sample.

The metal mandibular implant was constructed using the Renishaw AM400 SLM 3D printer (Renishaw plc, Wotton-under-Edge, Gloucestershire, UK). The implant material was a TiAlV alloy. Following 3D printing, the implant model was placed in the resin model of the mandible, and 2.7 × 12 mm screws (50-27412, Stryker, Kalamazoo, MI, USA) were locked into the wing plate to construct a model of the implant (Figure 4).

**Figure 5:**
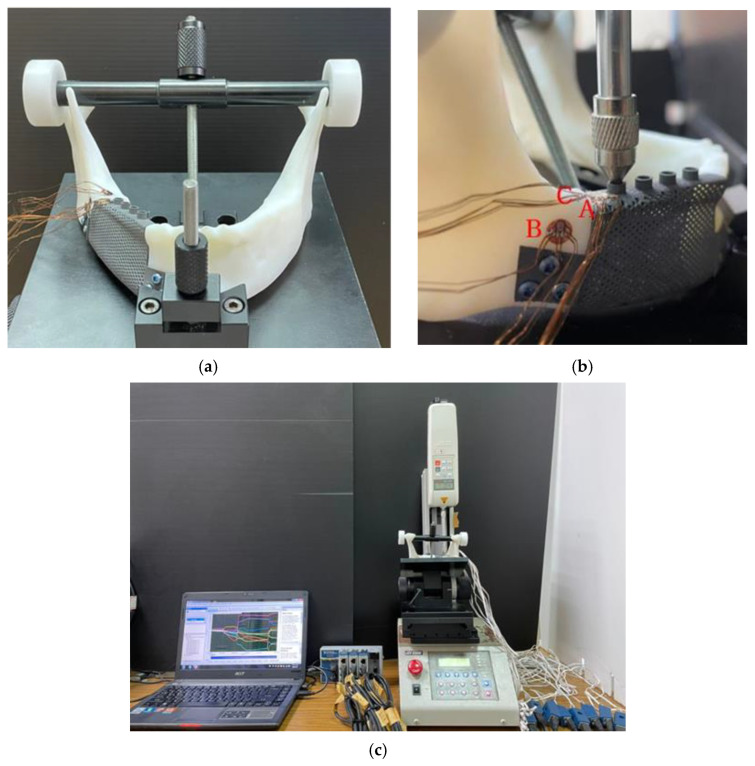
Photographs of (**a**) the experimental sample on the self-developed jig; (**b**) the positions of the strain gauge rosettes and their corresponding loading conditions (A indicates the implant body; B and C indicate the surrounding bone); and (**c**) equipment used for the in vitro experiments.

As displayed in Figure 5b, strain gauge rosettes (Kyowa Electronic Instruments Co., Ltd., Tokyo, Japan) comprising three independent strain gauges (εa, εb, and εc) were adhered to the implant body (designated as position A) and the surrounding bone (designated as positions B and C). The strain values at three locations were determined using a data acquisition system (CompactDAQ, National Instruments, Austin, TX, USA) and the associated software (LabVIEW SignalExpress 3.0, National Instruments, Austin, TX, USA).

The regions between the condylar and coronoid processes as well as the mental protuberance of the full jawbone model were fixed by installing a self-developed jig (Figure 5a) on the testing platform of a loading machine (JSV-H1000, Japan Instrumentation System, Nara, Japan). Regarding the loading conditions, the loading head axially pressed down on the abutment base of the right mandible at a speed of 1 mm per minute (Figure 5b). After each measurement had been repeated five times for each sample, the maximum (εmax) and minimum (εmin) principal strains of εa, εb, and εc were determined (Figure 5c).

All the measured data are presented as medians and interquartile ranges because of the small sample size. The Wilcoxon rank sum test was performed to compare the measurement data corresponding to the porous and nonporous implant designs. Analyses were conducted using SPSS version 19 (IBM, Armonk, NY, USA). The alpha value was 0.05.

## 3. Results

### 3.1. In Vitro Experiments

The results of analyses of the maximum principal strain (tensile strain) and the minimum principal strain (compressive strain) of the control sample and the sample with 1 mm circular holes are presented in Table 3.

As presented in Table 3 the control and experimental samples differed significantly (*p* < 0.05) in the maximum principal strain at positions A, B, and C. A significant between-sample difference was also observed between the minimum principal strain at positions A and C (*p* < 0.05). However, no significant difference in the minimum principal strain at location B was noted (*p* > 0.05).

### 3.2. FEA

**Figure 6:**
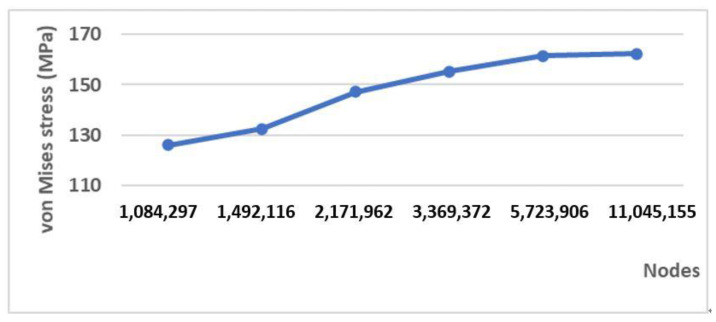
Results of the convergence test.

The results of the convergence analysis are shown in Figure 6. In line with expectations, a 0.4 mm element size for the implant and the surrounding bone mesh resulted in an error of 0.56% in the convergence results of the implant body.

#### 3.2.1. Von Mises Stress of the Implant Body

**Figure 7:**
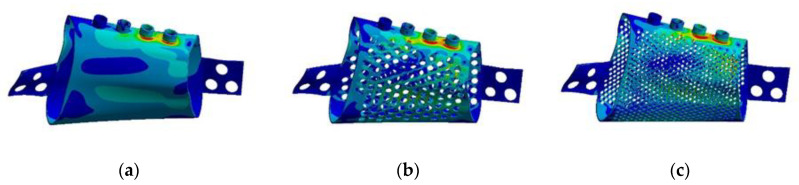

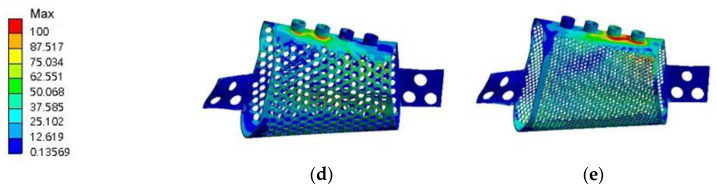
Von Mises stress distributions of the models in RMOL mode (**a**) without pores, (**b**) with circular holes 2 mm in diameter, (**c**) with circular holes 1 mm in diameter, (**d**) with hexagonal holes 2 mm in diameter, and (**e**) with hexagonal holes 1 mm in diameter.

**RMOL mode**. The peak stress of the control sample, 162.31 MPa, occurred around the base of abutments near the first and second molar areas (Figure 7). The peak stresses of the implants with 2 mm and 1 mm circular holes were 215 and 271 MPa, respectively, and the peak stresses of the implants with 2 mm and 1 mm hexagonal holes were 255 and 299 MPa, respectively. For mandibles with both circular and hexagonal holes, high stress was detected not only around the base of the abutments but also near the holes close to the base of the abutment (Figure 7).

**Figure 8:**
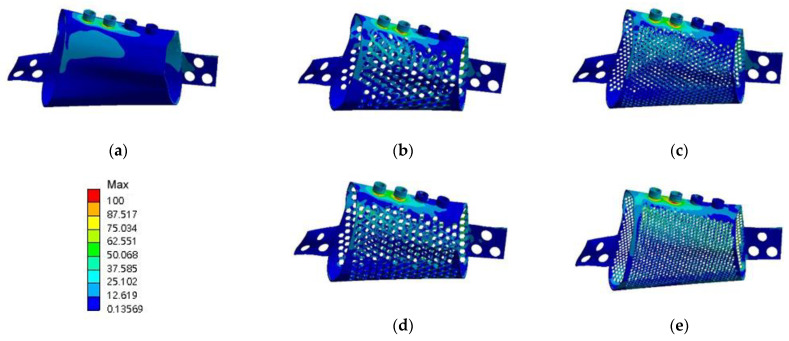
Von Mises stress distributions of the models in RGF mode (**a**) without pores, (**b**) with circular holes 2 mm in diameter, (**c**) with circular holes 1 mm in diameter, (**d**) with hexagonal holes 2 mm in diameter, and (**e**) with hexagonal holes 1 mm in diameter.

**RGF mode.** The peak stress of the control sample was 112.66 MPa. The peak stresses of the implants with 2 mm circular holes, 1 mm circular holes, 2 mm hexagonal holes, and 1 mm hexagonal holes were 141, 188, 247, and 332 MPa, respectively. The high-stress areas under the RMOL and RGF modes were similar (Figure 8).

#### 3.2.2. Von Mises Strain in the Bone around the Implant

**Figure 9:**
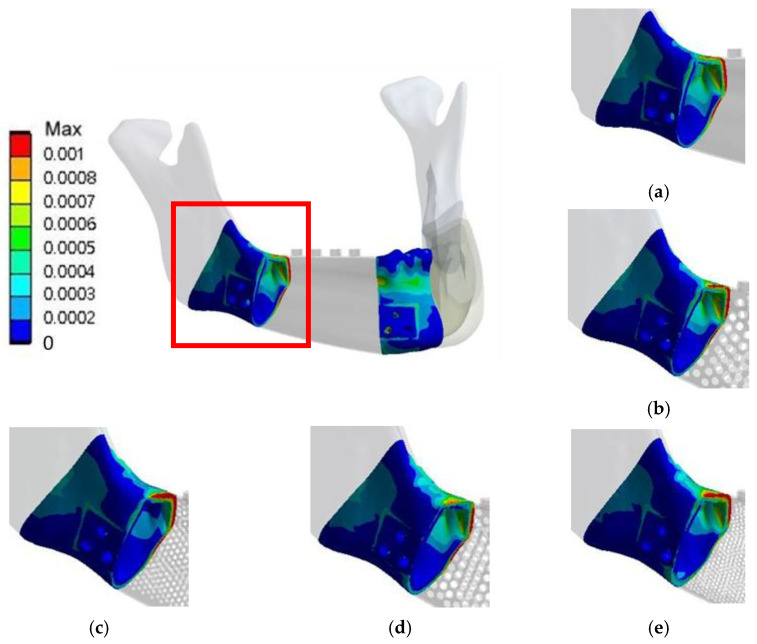
Distributions of von Mises strain in the bone of the models in RMOL mode (**a**) without pores, (**b**) with circular holes 2 mm in diameter, (**c**) with circular holes 1 mm in diameter, (**d**) with hexagonal holes 2 mm in diameter, and (**e**) with hexagonal holes 1 mm in diameter.

**RMOL mode**. The maximum von Mises strain in the bone around the control implant was 2664 µε. The maximum von Mises strains in the bone around the implants with 2 mm and 1 mm circular holes were 2648 and 2744 µε, respectively. The corresponding values for the implants with 2 mm and 1 mm hexagonal holes were 2678 and 2813 µε, respectively. High bone strain was observed near the connection area between the lingual side of the jawbone and the implant (Figure 9).

**Figure 10:**
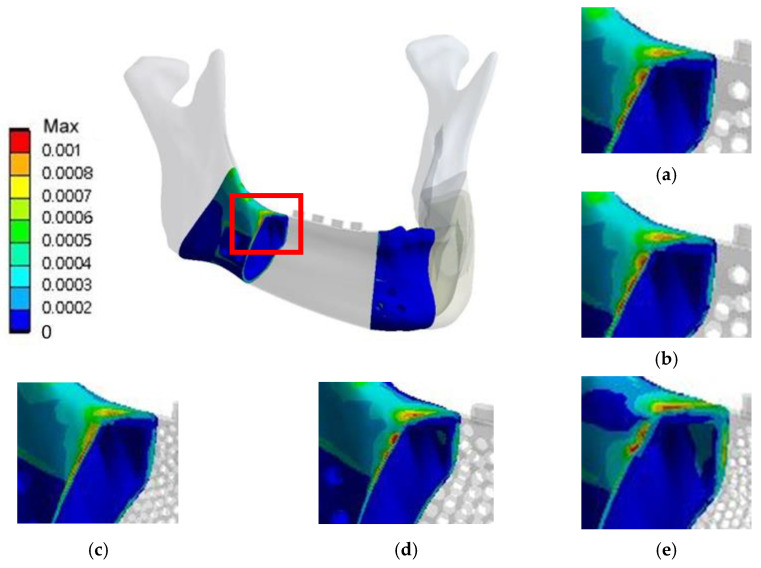
Distributions of von Mises strain in the bone of models the models in RGF mode (**a**) without pores, (**b**) with circular holes 2 mm in diameter, (**c**) with circular holes 1 mm in diameter, (**d**) with hexagonal holes 2 mm in diameter, and (**e**) with hexagonal holes 1 mm in diameter.

**RGF mode.** The maximum von Mises strain in the bone around the control implant was 1283 µε. The corresponding values for the implants with 2 mm circular holes, 1 mm circular holes, 2 mm hexagonal holes, and 1 mm hexagonal holes were 1250, 1161, 1354, and 1493 µε, respectively. The high-strain distributions of bone under the RMOL and RGF modes were similar (Figure 10).

## 4. Discussion

The results of the in vitro experiments demonstrate the influence of the porous design on the implant and on the surrounding bone. The minimum principal strain of the control sample at position A was relatively small. Regarding the strain values of the bone around the implant (positions B and C), compared with the control sample, the experimental sample (1 mm circular holes) had a larger maximum principal strain and a smaller minimum principal strain. Overall, the compressive strain of the control sample was smaller, but more compressive strain was transmitted to the surrounding bone, resulting in higher compressive strain in that area. The opposite trend was detected in the strain results of the experimental sample (1 mm circular holes); the compressive strain of the implant body was lower, whereas the compressive strain of the surrounding bone was higher. However, the maximum principal strain (i.e., tensile strain) in the bone surrounding the experimental sample (1 mm circular holes) was higher than that in the bone surrounding the control sample.

The porous samples had higher stress values in position A than the nonporous implants. However, the porous design made the stress distribution of the implant relatively uniform. Moiduddin et al. (2017) [23] evaluated the biomechanical performance of a self-designed bone plate and a porous implant for correcting mandibular defects. They reported that the porous design uniformized the stress distribution of the implant, preventing the concentration of stress in a specific area of the implant body.

In the present study, the stress values of the implants with hexagonal holes were higher than those of implants with round holes. A possible reason is that stress can accumulate in the angles of the hexagonal holes. However, under both the RMOL and RGF modes, the stress values of the five implant designs did not exceed the yield strength of Ti6Al4V (800 MPa) [23]. In other words, regardless of whether the implants were porous or nonporous, they were not at immediate risk of breakage.

The present results indicate that the porous design exerted no absolute influence on the strain value of the bone surrounding the implants. The strain values of the bone surrounding the control sample and the sample with 1 mm circular holes were comparable. However, if implants with hexagonal holes were used, the strain of the surrounding bone was higher. Furthermore, the smaller the size of the hole, the higher the strain in the bone around the implant. Frost’s mechanostat theory of bone [24,25] addresses the following four ranges of bone remodeling. (1) Disuse: when bone deformation is less than approximately 800 µε, bone resorption occurs, reducing bone quality and bone strength. (2) Adapted state: bone deformation is between approximately 800 µε and approximately 1500 µε. Bone mass and bone strength remain constant, and bone absorption is equal to bone formation. (3) Overload: after bone deformation exceeds approximately 1500 µε, bone growth increases bone quality and bone strength. (4) Pathologic fracture: when the bone deformation exceeds approximately 15,000 µε, the elasticity of bone deformation exceeds the maximum allowable value, resulting in fracture [26]. According to the present results, regardless of whether the occlusal mode was RMOL or RGF, the strain in the surrounding bone did not exceed 15,000 µε in any model, meaning that no risk of bone fracture was immediate. However, compared with that in RGF mode, bone strain in RMOL mode was in the overload stage (between 2648 and 2813 µε), potentially promoting bone growth in the surrounding bone contacted by the implant. In RGF mode, the strain in the surrounding bone strain was between 1161 and 1493 µε (adapted state), and the condition of the bone surrounding the implant could be maintained.

This study has some limitations. In the metal mandibular implant model, bone grafts were not placed in the hollow of the implant. The present analysis mainly simulated the biomechanical performance of the implants in the worst-case scenario, and the characteristics of bone materials were simplified to be homogeneous and isotropic, differing from real bone conditions. Furthermore, both the bone–screw interface and the bone plate–screw interface were bonded. This may not be the case in clinical contexts. Finally, animal experiments or preclinical studies are warranted to evaluate the feasibility of the implant models before their clinical application.

## 5. Conclusions

The conclusions are as follows. First, the porous implants had higher stress than the nonporous implants, but the value did not exceed the yield strength of Ti6Al4V (800 MPa), indicating that the porous implants were not at immediate risk of breakage. Second, the shape and size of the pores affected the stress value of the implant; specifically, implants with hexagonal holes had higher stress values than implants with circular holes. Finally, whether the implant was porous or nonporous, the strain in the surrounding bone was not consistent under differing occlusal conditions. Moreover, RMOL mode produced higher strain in the surrounding bone than RGF mode. According to Frost’s mechanostat theory of bone, this higher strain may promote improvements in bone quality and bone strength.

## Figures and Tables

**Table 1:** Material parameters of the model [19].

Material	Young’s Modulus (MPa)	Poisson’s Ratio
3D-printed titanium alloy	129,000	0.34
Titanium alloy	110,000	0.33
Cortical bone	13,400	0.3
Trabecular bone	790	0.3

**Table 2:** Magnitude of each muscular force under the RMOL and RFG loading conditions [21,22].

Clenching Tasks	Side	Direction	Muscular Force						Occlusal Force	
			SM	DM	MP	AT	MT	PT		
RMOL	Right	Force	137.1	58.8	146.8	115.3	63.1	44.6	Fx Fy Fz	−100
		Fx	−28.1	−32.1	71.4	−17.2	−14.0	−9.3		
		Fy	−57.4	21.0	−54.8	−5.1	31.5	38.1		
		Fz	121.2	44.5	116.1	114.0	52.8	21.1		
	Left	Force	114.2	49.0	104.9	91.6	64.1	29.5		
		Fx	23.6	26.7	−51.0	13.7	14.2	6.1		
		Fy	−47.9	17.5	−39.1	−4.0	32.0	25.2		
		Fz	101.0	37.1	83.0	90.5	53.6	14.0		
RGF	Right	Force	34.3	29.4	12.2	104.3	61.2	46.9	Fx Fy Fz	−100
		Fx	−7.1	−16.0	6.0	−15.5	−13.6	9.8		
		Fy	−14.4	10.5	−4.6	−4.6	30.6	40.1		
		Fz	30.3	22.3	9.7	103.0	51.2	22.2		
	Left	Force	51.4	21.2	132.8	11.1	5.7	4.5		
		Fx	10.6	11.6	−64.6	1.7	−1.3	−0.9		
		Fy	−21.5	7.6	−49.6	−0.5	2.9	3.9		
		Fz	45.4	16.1	105.1	10.9	4.8	2.2		

*Note:* SM, DM, MP, AT, MT, and PT refer to the superficial masseter, deep masseter, medial pterygoid, anterior temporalis, middle temporalis, and posterior temporalis, respectively. The occlusal forces were applied on the molar and premolar areas under the RMOL and RFG modes, respectively.

**Table 3:** Maximum and minimum principal strains of the control and experimental samples.

	Position		Control	Experiment	*p*
Max principal strain (μ strain)	A	Median	715.23	−90.61	0.021
		IQR	221.00	738.48	
	B	Median	1270.42	3114.37	0.021
		IQR	260.84	419.94	
	C	Median	480.41	1109.03	0.021
		IQR	89.44	2442.460	
Min principal strain (μ strain)	A	Median	−1525.53	−2246.89	0.021
		IQR	218.18	96.52	
	B	Median	−1240.42	−1077.21	0.083
		IQR	174.62	184.85	
	C	Median	−1497.91	−514.52	0.021
		IQR	148.19	461.84	

*Note*: IQR refers to interquartile range.

## Data Availability

The data that support the findings of this study are available from the corresponding author upon reasonable request.
